# Upregulation of *CASP9* through NF-κB and Its Target MiR-1276 Contributed to TNFα-Promoted Apoptosis of Cancer Cells Induced by Doxorubicin

**DOI:** 10.3390/ijms21072290

**Published:** 2020-03-26

**Authors:** Fei Zhou, Yun Li, Yisheng Huang, Jian Wu, Qinhan Wu, Hui Zhu, Jinke Wang

**Affiliations:** 1 School of Food Engineering and Biotechnology, Hanshan Normal University, Chaozhou 521041, China; zhoufei1200@163.com (F.Z.);; 2 State Key Laboratory of Bioelectronics, Southeast University, Nanjing 210096, China

**Keywords:** NF-κB, miR1276, CASP9, apoptosis

## Abstract

Under some conditions, nuclear factor-κB (NF-κB) has a pro-apoptotic role, but the mechanisms underlying this function remain unclear. This study demonstrated that NF-κB directly binds to *CASP9* and miR1276 in tumor necrosis factor α (TNFα)-treated HeLa and HepG2 cells. NF-κB upregulated CASP9 expression, whereas downregulated miR1276 expression in the TNFα-treated cells. The miR1276 repressed CASP9 expression in both cells. As a result, a typical NF-κB-mediated coherent feed-forward loop was formed in the TNFα-treated cells. It was proposed that the NF-κB-mediated loop may contribute to cell apoptosis under certain conditions. This opinion was supported by the following evidence: TNFα promoted the apoptosis of HeLa and HepG2 cells induced by doxorubicin (DOX). CASP9 was significantly upregulated and activated by TNFα in the DOX-induced cells. Moreover, a known inhibitor of CASP9 activation significantly repressed the TNFα promotion of apoptosis induced by DOX. These findings indicate that CASP9 is a new mediator of the NF-κB pro-apoptotic pathway, at least in such conditions. This study therefore provides new insights into the pro-apoptotic role of NF-κB. The results also shed new light on the molecular mechanism underlying TNFα-promotion of cancer cells apoptosis induced by some anticancer drugs such as DOX.

## 1. Introduction

Nuclear factor-κB (NF-κB) is an inducible transcription factor (TF) [1]. The NF-κB TF family consists of five members, namely p50, p52, p65/RelA, c-Rel and RelB, which can form various heterodimers or homodimers to bind to a DNA sequence motif known as the κB site [1]. After exposure of cells to inducers such as tumor necrosis factor α (TNFα), the inhibition of nuclear translocation and DNA-binding activity of NF-κB by IκB proteins is removed [1,2]. The activated NF-κB binds to κB sites in genomic DNA and regulates the transcription of its target genes in these cells [1]. In addition, NF-κB also regulates the expressions of microRNAs (miRNAs) at the transcriptional level [3]. miRNAs are critical regulators of gene expression, which act as post-transcriptional repressors by binding to the 3′untranslated regions (UTRs) of their target genes [3]. The interplay between NF-κB and its regulated miRNAs creates positive or negative feedback loops and regulatory networks, which can control cell fate, such as cell apoptosis [3]. 

Caspase (CASP) proteins are encoded by genes of the *CASP* family. These proteins are essentially aspartate-specific cysteine proteases that play a vital role in the induction, transduction and amplification of intracellular apoptotic signals [4,5]. Among these proteins, the CASP9 protein is a key initiator of the caspase cascade in the cell apoptotic process [4]. In the presence of apoptotic stimuli, procaspase 9 (the full-length CASP9 protein, fCASP9) forms an apoptosome complex with Apaf1 and cytochrome c in the cytoplasm, and this complex cleaves fCASP9 into active CASP9, named cleaved CASP9 (cCASP9). The cCASP9 protein then activates other caspases that induce cell apoptosis, such as CASP3 and CASP7 [5,6,7]. A previous study indicated that NF-κB directly binds and upregulates *CASP4* to facilitate cell apoptosis [8]. NF-κB also upregulates *Casp11* in LPS- and IFN-γ-induced mouse macrophages [9]. Under certain conditions, Casp11 can also activate CASP3, which lead to cells apoptosis [9]. However, it is unclear whether NF-κB regulates other *CASP* genes to play a role in cell apoptosis.

In general, NF-κB plays an anti-apoptotic role, for example, protection against TNFα-induced apoptosis [2,10,11,12]. However, there is growing evidence that NF-κB promotes apoptosis under certain conditions, e.g., in TRAIL- and FAS/CD95-mediated apoptosis [8,11], p53-induced apoptosis [12] and chemotherapeutic drug such as doxorubicin (DOX)-induced apoptosis [13,14,15]. NF-κB promotes these apoptotic processes by regulating several pro-apoptotic target genes. For example, NF-κB upregulates the target gene *CASP4*, which contributes to Fas-induced apoptosis [8]. NF-κB upregulates the target gene *PUMA* to promote DOX-induced apoptosis [15]. NF-κB upregulates the target gene *TRAIL*, which plays a key role in TNFα-enhanced apoptosis of breast cancer cells induced by DOX [13]. Therefore, the regulation of pro-apoptotic target genes by NF-κB is critical for the realization of the pro-apoptotic functions of this TF, but the exact molecular mechanism underlying NF-κB-promoting apoptosis is still poorly understood. 

In this study, we identify *CASP9* and miR1276 as two direct target genes of NF-κB in TNFα-treated HeLa cells and HepG2 cells. In these cells, NF-κB upregulates the expression of *CASP9* at both the mRNA and protein levels by directly binding to *CASP9*, but represses the expressions of miR1276 and its host gene *KLHL25*. We also find that miR1276 represses the expression of *CASP9*. Therefore, NF-κB can not only directly regulate *CASP9* by itself, but also indirectly regulates *CASP9* through miR1276. In other words, NF-κB can regulate *CASP9* via a coherent feed-forward loop (FFL) formed by itself and its direct target genes, *CASP9* and miR1276. We find that the regulation of *CASP9* through NF-κB-mediated FFL plays a pro-apoptotic role in the TNFα-promoted apoptosis of cancer cells induced by DOX. This study sheds new light on the molecular mechanisms underlying NF-κB-promoting apoptosis.

## 2. Results

### 2.1. NF-κB Binds to CASP9

We previously detected the global NF-κB-binding sites (BSs) of NF-κB RelA in TNFα-stimulated HeLa cells using the Chromatin immunoprecipitation-Sequencing (ChIP-Seq) technique [16]. Through a recent data analysis, we found that multiple BSs could be assigned to the *CASP9* gene. To the best of our knowledge, the gene encodes an important initiator caspase of cell apoptosis; however, there are no studies on whether this gene is a NF-κB-target gene. Based on the importance of *CASP9* for cell apoptosis and the current acknowledged anti-apoptotic function of NF-κB in cancer, we think it is necessary to study whether NF-κB regulates the *CASP9* gene, which may provide new insight into the potential pro-apoptotic role of this TF. 

**Figure 1:**
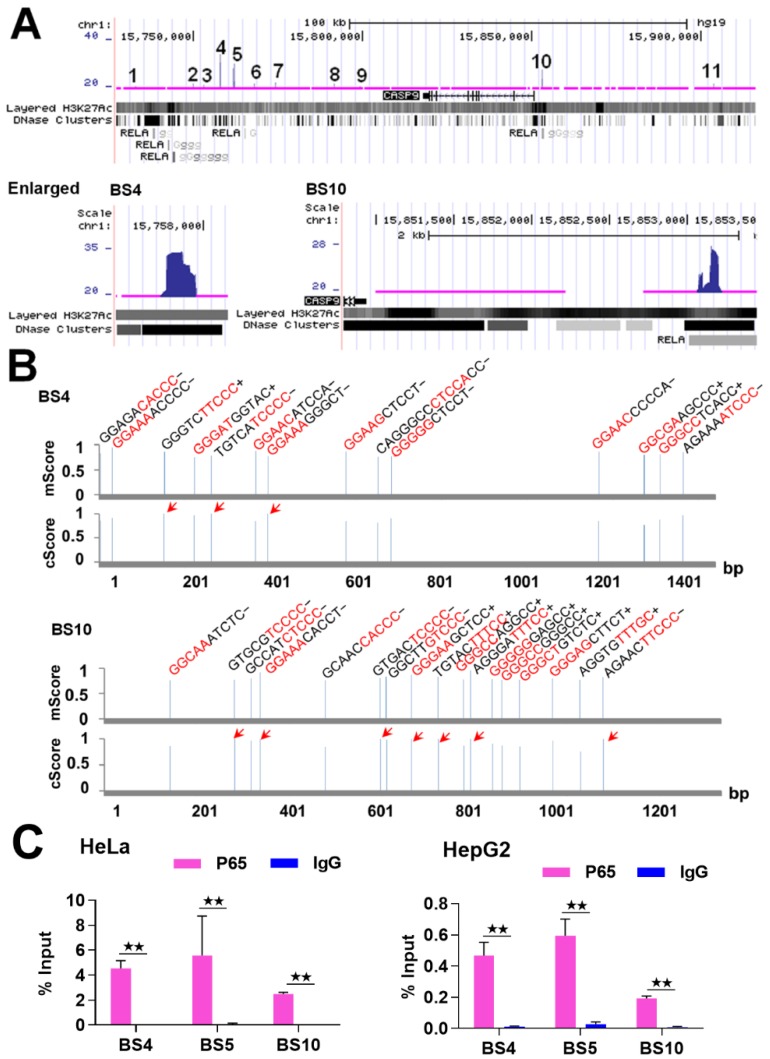
The binding of NF-κB to *CASP9* gene. (**A**) NF-κB-BSs (i.e., ChIP-Seq peaks; labeled with numbers) assigned to the *CASP9* gene in TNFα-treated HeLa cells. BS4 and BS10 are magnified in the image. (**B**) Predicted κBs in BS4 and BS10. The relative position, sequence and match score of κBs in the two BSs are shown. The five most conserved bases in the core match analysis are shown in red. The κBs with a core score of 1 are highlighted with arrows. mScore, matrix match score; cScore, core match score. (**C**) ChIP-qPCR detection of three BSs assigned to *CASP9* in TNFα-treated HeLa and HepG2 cells. The data are presented as the means ± SD from at least three replicates. The significance of the difference was analyzed by Student’s t-test. * *p* < 0.05; ** *p* < 0.01.

Given that NF-κB is a DNA-binding TF, it must bind to a gene to regulate its expression. Therefore, we first examined the binding of NF-κB to the *CASP9* gene. Similarly to other studies that found in vivo NF-κB-BSs using the ChIP-Seq technique [17,18], we found that NF-κB/RelA binds to the *CASP9* gene at multiple sites detected by ChIP-Seq in TNFα-treated HeLa cells (Table 1). Upon examination of these BSs (i.e., ChIP-Seq peaks) using the UCSC Genome Browser (Figure 1A), we noted that these BSs almost overlapped with the NF-κB/RelA-BSs found in TNFα-treated lymphoblastic cells by the Encyclopedia of DNA Elements (ENCODE) project [19], which indirectly indicates the reliability of these NF-κB-BSs found in TNFα-treated HeLa cells.

Because NF-κB binds to DNAs via specific DNA-binding sequences (i.e., κB) [20], the analysis of κB in the NF-κB-BSs can theorize whether NF-κB binds to these BSs directly or indirectly. Furthermore, mutation of the high-confidence κB predicted is necessary for the reporter gene assay. For this purpose, we then searched for potential κB in the NF-κB BSs assigned to the *CASP9* gene using the TRANSFAC program. The results revealed that each of these NF-κB-BSs contained multiple κBs (Table 1). For example, BS4 and BS10 contained 14 and 17 κBs, respectively, and there were some κBs in the both BSs with the highest core match score (Figure 1B). These data suggest that NF-κB directly binds to *CASP9* gene in the examined cells.

To further confirm that NF-κB binds to *CASP9* gene, we subsequently detected these NF-κB-BSs through ChIP-qPCR assay. We first re-performed the ChIP experiment with TNFα-treated HeLa cells as in our previous ChIP-Seq study, and then detected BS4, BS5 and BS10 (Table 1 and Figure 1) with the input and ChIPed DNAs through qPCR assay. The results revealed that these BSs were significantly enriched by the RelA antibody compared with normal IgG (Figure 1C). This finding indicates that NF-κB truly binds to the *CASP9* gene in these cells. By performing similar experiments with TNFα-treated HepG2 cells, we also found that NF-κB bound to the three BSs in HepG2 cells (Figure 1C). Together, these data demonstrate that NF-κB directly binds to the *CASP9* gene in the examined cells.

**Figure 2:**
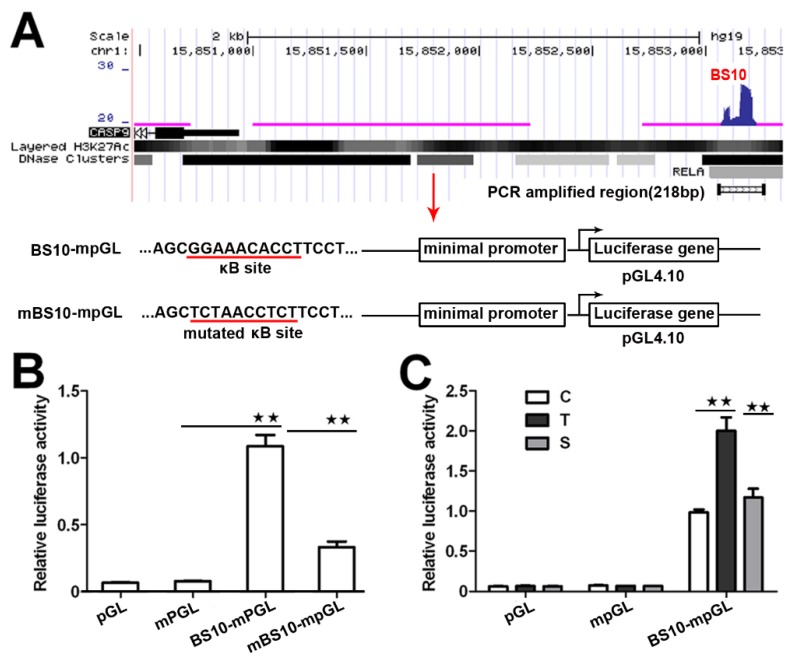
Transcriptional activity of NF-κB-BSs assigned to *CASP9*. (**A**) Schematic showing the luciferase constructs prepared with the pGL4.10 basic vector (pGL). mpGL, pGL with a minimal promoter sequence; BS10-mpGL, mpGL with a 218-bp sequence of BS10; mBS10-mpGL, BS10-mpGL with a mutated κB site. (**B**,**C**) Relative transcriptional activity in HeLa cells determined through a dual-luciferase reporter assay. C, control cells; T, TNFα-treated cells; S, RelA siRNA and TNFα co-treated cells. The data are presented as the means ± SEM from three independent experiments with three replicates in each test. The significance of the difference was analyzed by one-way ANOVA. * *p* < 0.05; ** *p* < 0.01.

To investigate whether these BSs have a transcriptional function, we next analyzed the transcriptional activity of these NF-κB-BSs using a reporter construct. We prepared a reporter construct by cloning the center sequence of BS10 (Figure 2A) into the pGL4.10 plasmid with a minimal promoter. We also prepared a mutated reporter construct, in which a predicted κB in BS10 with a match score over 0.95 was replaced with a reported scramble sequence (5′-TCTAACCTCT-3′) without κB [21]. We then evaluated their transcriptional activity through a dual-luciferase reporter assay. The results indicate that the BS10-containing construct significantly increases the relative luciferase activity in the transfected HeLa cells (Figure 2B). However, the mutated construct markedly attenuated its transcriptional activity (Figure 2B). Moreover, the luciferase activity of the BS10-containing construct was increased by TNFα, whereas it was repressed by RelA siRNA. This finding demonstrates that the transcriptional activity of the BS10-containing construct is dependent on the cellular NF-κB activity (Figure 2C). These data suggest that the binding of NF-κB to the *CASP9* gene may function in the control of *CASP9* expression.

### 2.2. NF-κB Directly Regulates CASP9 Expression

To further confirm whether the NF-κB-DNA binding observed in HeLa cells and HepG2 cells is functional, we analyzed *CASP9* mRNA expression. The cellular NF-κB activity of HeLa cells was manipulated through TNFα (NF-κB activator) stimulation and RelA siRNA (NF-κB inhibitor) interference as in our previous study [22]. Signal Silence Control siRNA-treated HeLa cells were used as controls. 

**Figure 3:**
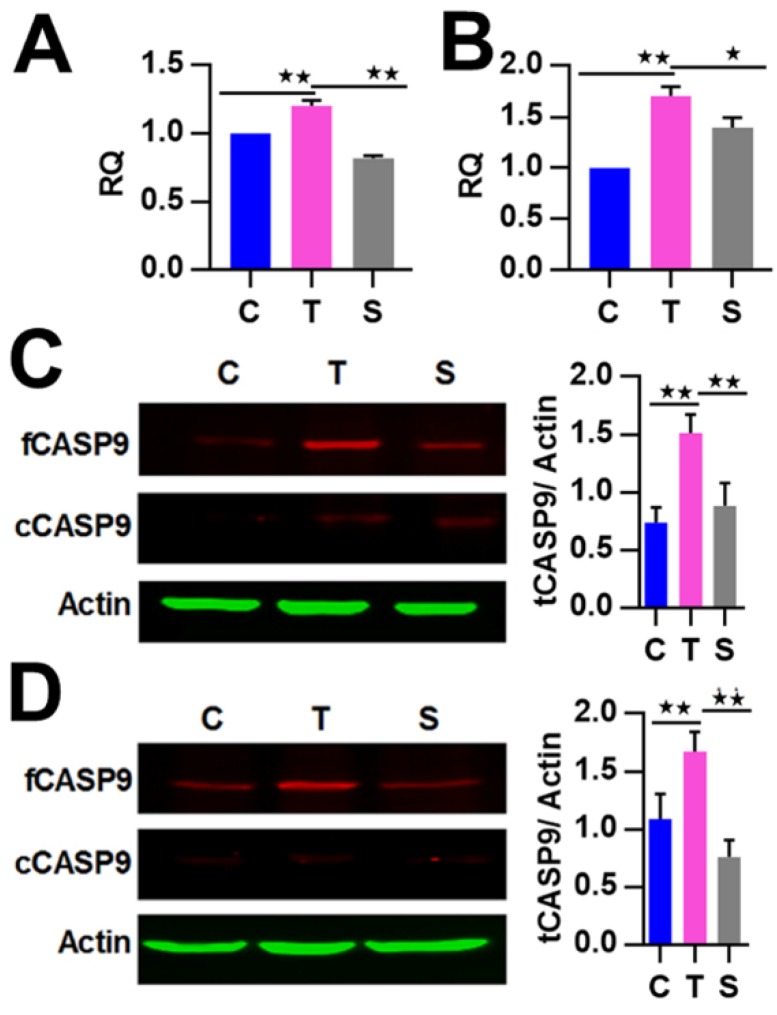
The regulation of *CASP9* by NF-κB. (**A**,**B**) Expression of *CASP9* mRNA in HeLa cells (**A**) and HepG2 cells (**B**). The data are presented as the means ± SEM from three independent experiments with three replicates in each test. *GAPDH* was used as the endogenous control to normalize *CASP9* expression. Relative quantification (RQ) is the *CASP9* expression normalized to *GAPDH* expression in each sample and presented relative to the control cells. (**C**,**D**) Expression of CASP9 protein in HeLa (**C**) and HepG2 (**D**) cells. The signal intensity of full-length CASP9 (fCASP9) and cleaved CASP9 (cCASP9) proteins were quantified with Image J software and normalized to that of the actin. The normalized signal intensity of fCASP9 plus that of cCASP9 was represented as normalized total CASP9 (tCASP9) signal that was visualized by a histogram at the right of blot images. The data are presented as the means ± SD from three independent experiments. C, control cells; T, TNFα-treated cells; S, RelA siRNA and TNFα co-treated cells. The significance of the difference was analyzed by one-way ANOVA. * *p* < 0.05; ** *p* < 0.01.

We then detected and found that the mRNA expression of *CASP9* in HeLa cells was significantly increased by TNFα stimulation and inhibited by RelA siRNA (Figure 3A), which is consistent with our previous data obtained from Genechip assay (Figure S1A). Similar regulation of *CASP9* by NF-κB was also found in HepG2 cells (Figure 3B). These results indicated that NF-κB can regulate the expression of *CASP9* at the mRNA level. We next detected the regulation of CASP9 by NF-κB at the protein level through Western blot (WB) assay. The intracellular CASP9 is mainly composed of cleaved CASP9 (cCASP9) and full length CASP9 (fCASP9, i.e., procaspase 9) without being completely cleaved. The abundance of total CASP9 (tCASP9) is the sum of fCASP9 and cCASP9. As a result of WB assay, the tCASP9 proteins were significantly increased by TNFα stimulation but repressed by RelA siRNA in the both cells (Figure 3C,D). These results indicate that NF-κB regulates the expression of CASP9 at the protein level. Together, these data demonstrate that NF-κB can control the expression of *CASP9*. In combination with the binding properties of NF-κB checked to this gene, this finding indicates that *CASP9* is a direct target gene of NF-κB and that the activation of NF-κB can lead to the upregulation of the gene. 

### 2.3. NF-κB Indirectly Regulates CASP9 Expression

We preliminarily identified miR1276 as a target miRNA of NF-κB and *CASP9* as a target of this miRNA in TNFα-treated HeLa cells using the global data obtained through ChIP-Seq, Gene chip and miRNA-Seq [23]. Based on the above-presented high-throughput data, we reasoned that NF-κB might also indirectly regulate the expression of *CASP9* via miR1276; in other words, *CASP9* may also be an indirect target gene of this TF. NF-κB was found to repress the expression of miR1276 in our previous study [23]. Therefore, we aimed to verify the NF-κB-mediated regulation of miR1276 and the miR1276-mediated regulation to *CASP9*, which is critical for understanding the biological role of this TF. 

**Figure 4:**
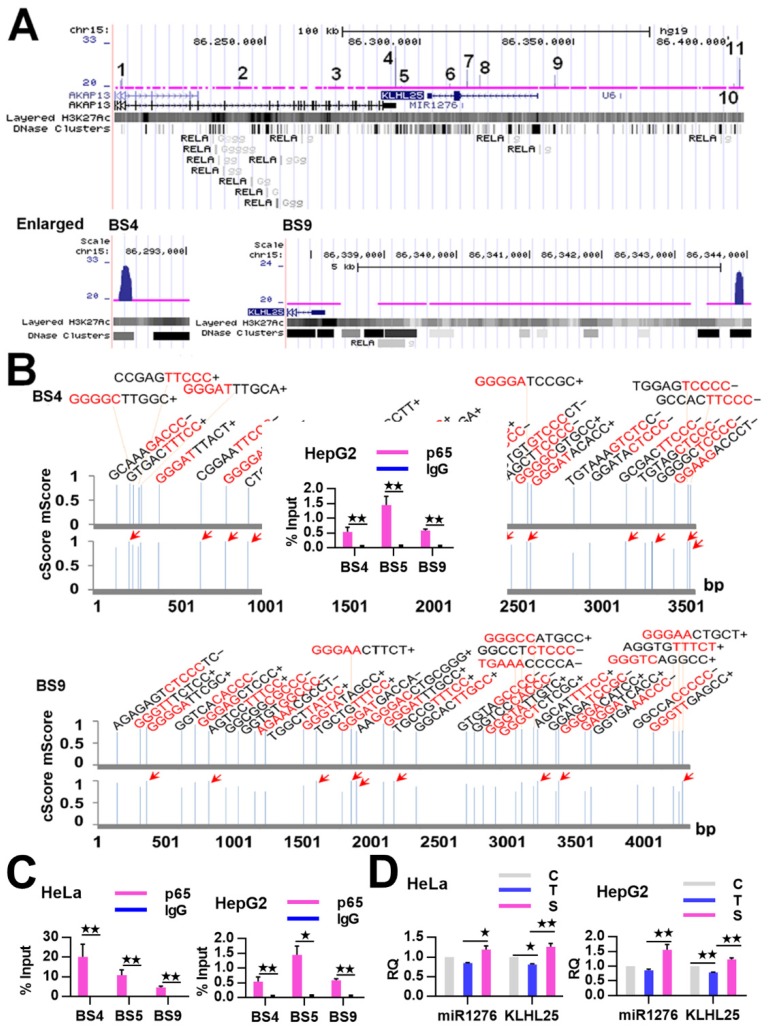
Binding and regulation of *KLHL25/miR1276* by NF-κB. (**A**) NF-κB-BSs (i.e., ChIP-Seq peaks; labeled with numbers) assigned to the *KLHL25*/*miR1276* genes in TNFα-treated HeLa cells. The magnified BS4 and BS9 are put on the bottom. (**B**) The predicted κBs in BS4 and BS9. The relative position, sequence and match score of κBs in the BSs are shown. Five most conserved bases in the core match analysis are shown in red. The κBs with a core score of 1 are highlighted with arrows. mScore, matrix match score; cScore, core match score. (**C**) ChIP-qPCR detections of three BSs assigned to the *KLHL25/ miR1276* genes in TNFα-treated HeLa and HepG2 cells. The data are presented as the means ± SD from at least three replicates. The significance of the differences was analyzed by Student’s t-test. * *p* < 0.05; ** *p* < 0.01. (**D**) Expression of miR1276 and *KLHL25* in HeLa cells and HepG2 cells. *GAPDH* or *U6* was used as the endogenous control in the qPCR detection to normalize the target genes. Relative quantification (RQ) is the expression of detected gene normalized to that of *GAPDH* or *U6* in each sample and presented relative to the control cells. The data are presented as the means ± SEM from three independent experiments with three replicates in each test. C, control cells; T, TNFα-treated cells; S, RelA siRNA and TNFα co-treated cells. The significance of the differences was analyzed by one-way ANOVA. * *p* < 0.05; ** *p* < 0.01.

By reanalyzing our ChIP-Seq data previously obtained from TNFα-treated HeLa cells [16], we found 11 high-confidence NF-κB-BSs (FE over 20) assigned to the miRNA1276 gene and its host gene *KLHL25* (Table 2). Similarly, we found that these BSs are closely related to the NF-κB/RelA-BSs found in TNFα-treated lymphoblastic cells by the ENCODE project (Figure 4A). The bioinformatic analysis revealed multiple κBs in each of these BSs (Table 2). For example, BS4 and BS9 harbored as many as 28 and 35 canonical κB sites, respectively (Figure 4B and Table 2), and some κBs had the highest core match score (1) (Figure 4B). We then confirmed these NF-κB BSs by examining some of them (BS4, BS5 and BS9) through ChIP-qPCR assay. The results revealed that the detected BSs were significantly enriched in TNFα-treated HeLa cells and HepG2 cells by the anti-RelA Ab compared with IgG (Figure 4C). The results of ChIP-qPCR demonstrated that the detected BSs were notably bound by NF-κB in TNFα-treated HeLa cells and HepG2 cells (Figure 4C). Together, these data demonstrate that NF-κB directly binds to the *KLHL25/miRNA1276* genes in the examined cells. 

We next investigated whether NF-κB controls the expression of miR1276 by binding to it. To this end, we detected the expression of miR1276 in HeLa and HepG2 cells, whose NF-κB activity were successfully manipulated as in our previous study [22]. As a result, we found that the expressions of miR1276 in these cells were significantly decreased by TNFα stimulation but increased by RelA siRNA treatment (Figure 4D). The qRT-PCR results revealed that NF-κB controls the expression of miR1276 in HeLa and HepG2 cells. To indirectly validate the finding that NF-κB controls miR1276 expression, we simultaneously detected the expression of the miR1276 host gene, *KLHL25*, in the HeLa and HepG2 cells through qRT-PCR assay. The results showed that NF-κB similarly controls the expression of *KLHL25* in these cells (Figure 4D). These qRT-PCR results are consistent with our previous results obtained through the miR-Seq and Genechip analysis of TNFα-treated HeLa cells (Figure S1B). Together, these data revealed that NF-κB directly downregulated miR1276 in both the examined cells.

**Figure 5:**
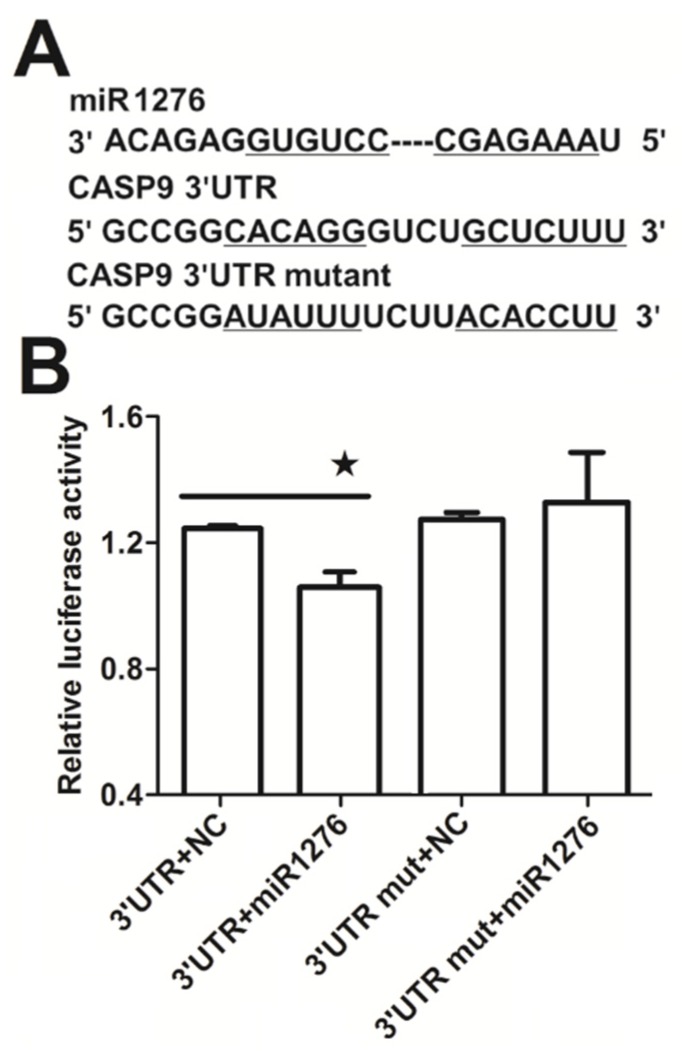
MiR1276-specific binding to the 3ʹUTR of *CASP9*. (**A**) Binding site in 3′UTR of the *CASP9* mRNA predicted by the TargetScan online program. (**B**) Relative luciferase activity analyzed by the dual-luciferase reporter assay in HeLa cells. The psiCHECK2-*CASP9* 3′UTR (3′UTR) or psiCHECK2-*CASP9* 3′UTR mutant (3ʹUTR mut) was co-transfected into HeLa cells with miR1276 expression plasmid (miR1276) or its negative control plasmid (NC). Results are presented as means ± SEM from three independent experiments with at least three replicates in each test. 3′UTR+NC vs. 3′UTR+miR1276 were analyzed with the Student’s t-test. * *p* < 0.05.

**Figure 6:**
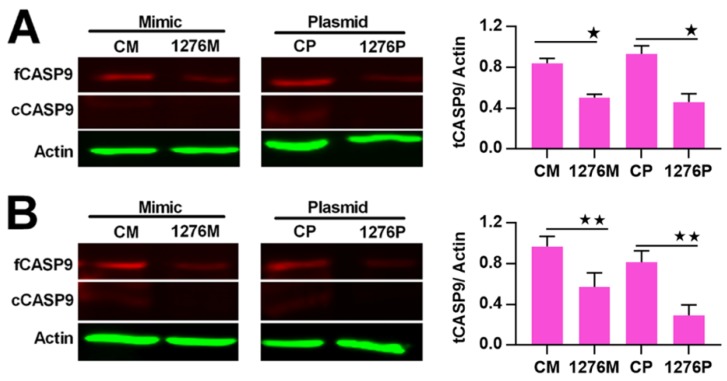
Regulation of CASP9 by miR1276 at the protein level. (**A**) and (**B**) Expression of CASP9 protein in HeLa cells (A) and HepG2 cells(B) transfected with miR1276 mimic or expression vector. The data are presented as the means ± SD from three independent experiments. fCASP9, cCASP9, tCASP9 and the calculation of tCASP9/actin; see Figure 4. CM, control mimic; 1276M, miR1276 mimic; CP, control plasmid; 1276P, miR1276 expression plasmid. The significance of the differences was analyzed by Student’s t-test. * *p* < 0.05; ** *p* < 0.01.

It is well known that miRNA plays a regulatory role in gene expression. We previously found that miR1276 can repress the transcription of *CASP9* in HeLa and HepG2 cells through qRT-PCR assay [23]. These results are consistent with the bioinformatic analysis, in which we analyzed the target sites of miR1276 in the *CASP9* mRNA (NM_001229) by various programs, including miRwalk, TargetScan and DIANA-microT-CDS. The bioinformatic results indicated that the 3′ UTR of *CASP9* mRNA contained 1, 1 and 6 potential miR1276 binding sites predicted by miRwalk, TargetScan and DIANA-microT-CDS programs, respectively (Table 3). To validate miR1276 binding to these predicted sites, we cloned the 3′UTR of *CASP9* containing the miR1276 binding site predicted by TargetScan programs into a luciferase reporter construct (psiCHECK2), in addition to a mutated *CASP9* 3′ UTR (Figure 5A). Relative luciferase activity of psiCHECK2-CASP9 3′UTR was significantly decreased by the miR1276-expressed plasmid compared with the negative control (NC) plasmid (Figure 5B). Furthermore, when the binding site of miR1276 was mutated, the relative luciferase activity was not decreased by the miR1276-expressed plasmid compared with NC plasmid (Figure 5B). These results indicated that miR1276 specifically bound to the 3′UTR of *CASP9*. To further confirm this regulation, we also investigated the effect of miR1276 on CASP9 protein. The results showed that the intracellular tCASP9 protein was significantly decreased by both miR1276 mimics and its overexpressed plasmid in HeLa and HepG2 cells (Figure 6A,B). These results demonstrated that miR1276 downregulated the expression of *CASP9* at both mRNA and protein levels in the detected cells. In other words, *CASP9* is a true target gene of miR1276.

The above findings show that NF-κB can directly and indirectly control *CASP9* expression at both the mRNA and protein levels in TNFα-treated HeLa and HepG2 cells. Essentially, NF-κB can indirectly enhance *CASP9* expression by directly repressing the expression of miR1276. Therefore, a coherent FFL forms between NF-κB and its two target genes, miR1276 and *CASP9*, and this NF-κB-mediated FFL upregulates the expression of *CASP9*. 

### 2.4. The Regulation of CASP9 by NF-κB-mediated FFL Contributes to the TNFα Promotion of DOX-Induced Apoptosis

**Figure 7:**
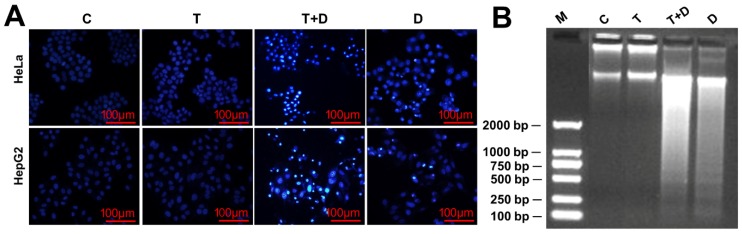
Doxorubicin (DOX)-induced apoptosis with or without TNFα-cotreatment. (**A**) The morphological characteristics of apoptotic cells. The cells were stained with 4′,6-diamidino-2-phenylindole (DAPI) and observed by fluorescence microscopes. (**B**) The DNA fragments of apoptotic cells were shown by agarose gel electrophoresis. C, control cells; T, TNFα-treated cells; T+D, TNFα &DOX-treated cells; D, DOX-treated cells.

**Figure 8:**
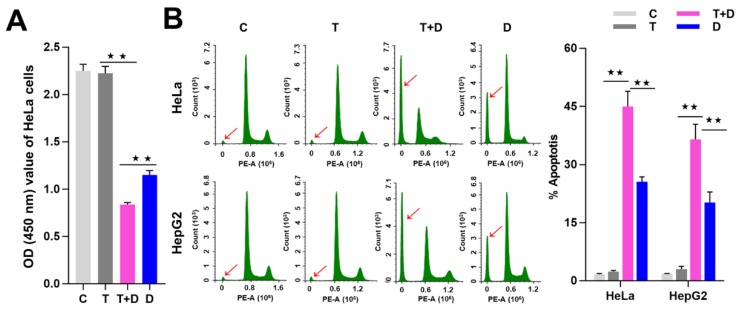
TNFα promotion of apoptosis induced by DOX. (**A**) Detection of the cell viability by CCK8 assay. (**B**) Detection of cell apoptosis by flow cytometry (FCM) assay. The sub-G1 peaks of FCM assay were labeled with red arrows. The average apoptotic rates of three FCM detections were shown by histogram. The data are presented as the means ±SD from three independent experiments. C, control cells; T, TNFα-treated cells; T+D, TNFα&DOX-treated cells; D, DOX-treated cells. The significance of the difference was analyzed by one-way ANOVA. * *p* < 0.05; ** *p* < 0.01.

Since CASP9 has the function of initiating apoptosis, we ask what effect does upregulation of CASP9 by the NF-κB-mediated FFL have on apoptosis? The upregulation of CASP9 by NF-κB-mediated FFL was demonstrated in TNFα-treated HeLa and HepG2 cells, but TNFα alone did not obviously induce apoptosis of HeLa and HepG2 cells (Figure 7 and Figure 8). A previous study showed that TNFα promotes the DOX-induced apoptosis of breast cancer cells [13]. The molecular mechanisms underlying the promotion effect of TNFα on DOX-induced apoptosis thus needs to be explored. We therefore speculated that the upregulation of CASP9 by NF-κB-mediated FFL might contribute to this biological process. To verify the hypothesis, we first examined the effect of TNFα on DOX-treated HeLa and HepG2 cells. As expected, microscopic observation and DNA laddering assay showed DOX alone or TNFα&DOX-induced apoptosis of HeLa cells and HepG2 cells (Figure 7). A quantitative analysis of cell viability (CCK8 assay) showed TNFα significantly promoted DOX-inhibited survival of HeLa cells (Figure 8A). Similarly, flow cytometry (FCM) assay also revealed that TNFα significantly promoted DOX-induced apoptosis of HeLa cells and HepG2 cells (apoptotic cells with sub-G1 DNA content); in other words, TNFα&DOX-treated cells had the highest apoptotic ratio (Figure 8B). 

**Figure 9:**
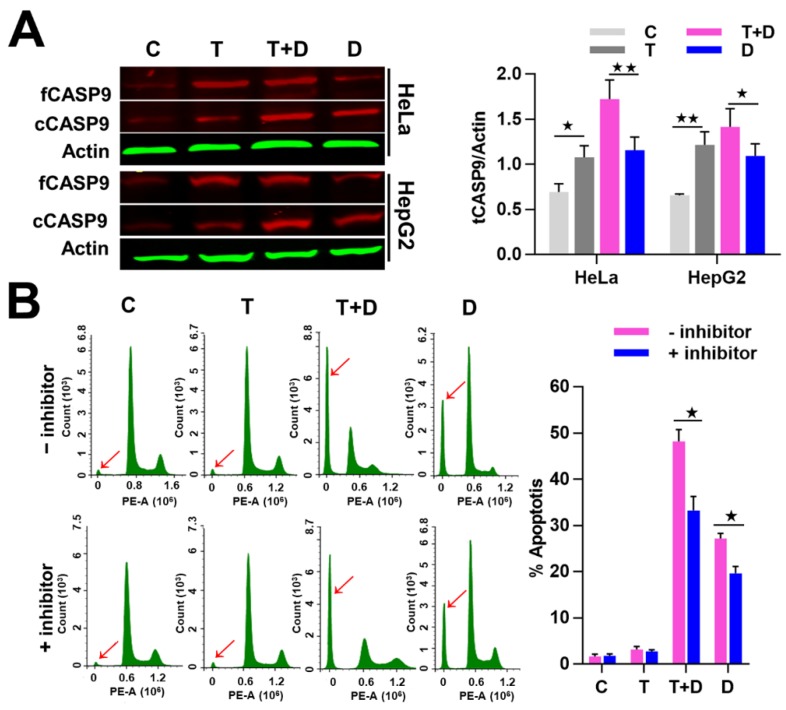
Contribution of NF-κB-upregulated CASP9 to TNFα promotion of apoptosis induced by DOX. (**A**) Expression of CASP9 protein. The data are presented as the means ±SD from three independent experiments. fCASP9, cCASP9, tCASP9 and total CASP9; see Figure 4. The significance of the difference was analyzed by one-way ANOVA. * *p* < 0.05; ** *p* < 0.01. (**B**) Effect of CASP9 inhibitor on cell apoptosis. Cells pretreated with 50 µM CASP9 inhibitor for 30 min were treated with TNFα, DOX, or TNFα&DOX for 12 h. The sub-G1 peaks obtained from the FCM assay are labeled with red arrows. The data are presented as the means ±SD from three independent FCM detections. The significance of the difference was analyzed by Student’s t-test. * *p* < 0.05; ** *p* < 0.01. C, T, T + D, D; see Figure 7.

To investigate whether the promoted effect of TNFα on DOX-induced apoptosis is related to CASP9, we then examined the activation of CASP9 protein in the negative control cells and cells treated with TNFα, DOX or TNFα&DOX through WB assay. As expected, the negative control and TNFα-treated cells did not undergo apoptosis and showed the lowest cCASP9 (active CASP9) level, the DOX-treated apoptotic cells showed a modest cCASP9 level and the TNFα&DOX-treated apoptotic cells with the highest apoptotic ratio showed the highest cCASP9 level in the examined cells (Figure 9A). We consequently analyzed the expression of tCASP9 protein in these cells. In line with the above findings, TNFα increased the tCASP9 in HeLa and HepG2 cells. However, the increased tCASP9 was not confined to TNFα alone-treated cells. Moreover, TNFα significantly increased the tCASP9 protein level in the TNFα&DOX-treated cells compared with that detected in the DOX-treated cells (Figure 9A). The detected increase of tCASP9 protein expression was not only consistent with the increased cCASP9 but was also in accordance with the increased apoptotic rate of HeLa cells and HepG2 cells (Figure 8). The highest tCASP9 protein level was found in TNFα&DOX-treated cells. These data indicated that the severe cell apoptotic events in TNFα&DOX-treated cells were correlated with upregulation of tCASP9. The up-expressed tCASP9 level increased the active CASP9 protein to initiate apoptosis. Clearly, the upregulation of CASP9 protein by NF-κB-mediated FFL was positively correlated with TNFα&DOX-induced severe apoptosis of cells. To validate the contribution of CASP9 to this cell apoptosis process, we also treated HeLa cells with a specific CASP9 inhibitor. As a result, we found that the CASP9 specific inhibitor significantly repressed the apoptosis of HeLa cells induced by the TNFα&DOX and DOX treatments (Figure 9B).

**Figure 10:**
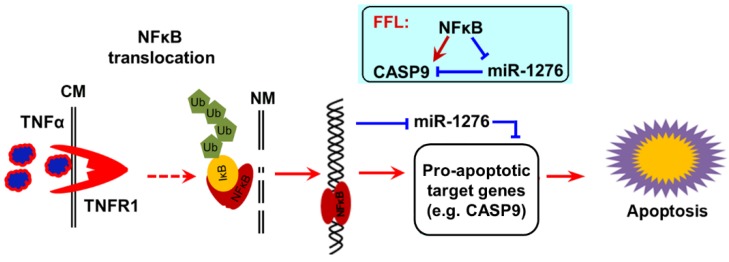
The scheme of NF-κB pro-apoptotic function. After activation by TNFα, NF-κB enters the cell nucleus, where it directly upregulates a pro-apoptotic target gene *CASP9* and directly downregulates a target miRNA miR1276. *CASP9* is a target gene of miR1276. A NF-κB-mediated feed-forward loop (FFL) is consequently established (inset). The up-expressions of pro-apoptotic target genes of NF-κB contributed to NF-κB-involved apoptosis under certain conditions.

Collectively, our findings strongly support a previous study which found that TNFα increases cytotoxicity of anticancer drugs such as DOX to cancer cells [13,24], because TNFα is a strong activator of NF-κB signal. We concluded that TNFα activates NF-κB activity in cancer cells, which in turn upregulates the pro-apoptotic target gene *CAPS9* at the mRNA level and protein level by direct transcriptional regulation, and indirectly decreases the expression of CASP9 repressor miR1276. Therefore, the up-expression of CAPS9 contributes to apoptosis of cancer cells, at least in the process of apoptosis induced by TNFα&DOX (Figure 10). 

## 3. Discussion

NF-κB is an inducible DNA-binding TF [1]. TNFα is one of the strong inducers of NF-κB and after inducing by TNFα, the activated intranuclear NF-κB binds and regulates a series of target genes to play an important role in physiological and pathological process [1,22]. Generally, NF-κB has an anti-apoptotic role, such as protection against TNFα-induced apoptosis [2,10,11,12]. However, there is mounting evidence that NF-κB can paradoxically suppress and promote apoptosis in response to TNFα. NF-κB has been found to promote TNFα-involved cell apoptosis via regulating pro-apoptotic genes including *p53*, *TRAIL*, *PUMA* and *FAS* [10,12,13,25,26]. Previous studies have shown that TNFα increases the efficiency of the anticancer chemotherapeutic drug DOX [13,24,27]. A combined treatment of TNF-α and DOX is a promising chemotherapeutic strategy to overcome cancer. Therefore, the molecular mechanism underlying the processes remains to be further explored. 

This study identified *CASP9* and *miR1276* as two direct target genes of NF-κB in TNFα-treated cells. The analysis of our previous NF-κB/RelA ChIP-Seq data [16] demonstrated that NF-κB binds to the *CASP9* gene, particularly to its promoter region, in TNFα-treated cells. Additionally, the investigation of κB sites and ChIP-qPCR further confirmed the direct binding of NF-κB to *CASP9* in TNFα-treated HeLa and HepG2 cells. Moreover, a reporter gene assay showed that the NF-κB-BSs assigned to the *CASP9* gene have a transcriptional function. We then demonstrated that NF-κB upregulated *CASP9* at both the mRNA and protein levels in TNFα-treated HeLa and HepG2 cells. These findings allowed us to identify *CASP9* as a positive direct target gene of NF-κB in TNFα-treated cells. In addition, we further validated an alternative indirect regulatory pathway through which NF-κB indirectly regulates *CASP9* expression. It was found that NF-κB directly bound to miR1276 and repressed its expression, whereas miR1276 repressed the expressions of CASP9 at the protein level in both HeLa and HepG2 cells. Therefore, we regarded miR1276 as a negative direct target gene of NF-κB through which NF-κB indirectly maintains the upregulations of *CASP9*. Consequently, a coherent FFL is established among NF-κB and its two target genes, miR1276 and *CASP9*, and this loop can upregulate the mRNA and protein levels of *CASP9* in TNFα-treated cells. Upregulations of CASP9 resulted in apoptosis which was demonstrated in diverse cell types, such as A-172 cells [28], Panc-1 cells [29], primary pituitary cells [30] and HeLa cells [30]. Therefore, the upregulation of *CASP9* by the NF-κB-mediated FFL is critical for apoptosis.

In its activated form, the cCASP9 can activate downstream caspases, including CASP3 and CASP7, which can result in cell apoptosis. CASP9 thus functions as a key initiator caspase in apoptosis [4,5,6,7]. The upregulations of CASP9 can markedly induce the apoptosis of diverse cells [28,29,30]. In the present study, we showed that TNFα alone can induce the upregulation of CASP9 through a NF-κB-mediated FFL, but that it did not induce the apoptosis of the detected cells at the dose of 30 ng/mL. This observation was consistent with previous findings that low doses (10, 20 or 50 ng/mL) of TNFα have no effect on cell survival [13,24,31]. NF-κB was therefore considered as having an anti-apoptotic role in TNFα signaling [10,11]. However, TNFα promotes the apoptosis of cells induced by chemotherapeutic drugs [13,24,31]. The pro-apoptotic target genes of NF-κB induced by TNFα were considered to play a critical role in the process of the increased apoptosis, such as *TRAIL* [13]. We also observed that TNFα significantly promoted the apoptosis of HeLa and HepG2 cells induced by DOX. This study, in conjunction with other observations [13,24,31], therefore indicated that TNFα can serve as a sensitizer to conventional chemotherapeutic agents to kill cancer cells by promoting their apoptosis. However, the molecular mechanism underlying this process remains unclear. In this study, NF-κB-dependent up-expression of pro-apoptotic CASP9, as said above, was found during the process of the promotion of apoptosis. Based on previous reports that up-expressions of CASP9 resulted in apoptosis [28,29,30], we hypothesized that the up-expressions of CASP9 by NF-κB-mediated FFL plays a critical role in the TNFα-induced sensitization to DOX. It may be that due to a degree of tolerance of tumor cells to CASP9, the up-expressed CASP9 did not induce apoptosis in TNFα alone-treated cells; however, it induced more severe apoptosis in TNFα&DOX-treated cells with the highest CASP9 protein level. It is likely that the alteration of CASP9 mediated by NF-κB is associated with cell apoptosis. This opinion is supported by our previous study. The study found that the inhibition activity of CASP9 by overexpression of an NF-κB inhibitor, HSCO, blocked the apoptosis of mouse NIH/3T3 fibroblasts induced by DOX and etoposide [32]. Besides, it is further supported by our finding that a CASP9 inhibitor attenuated TNFα-mediated promotion of apoptosis induced by DOX. Therefore, the TNFα-induced upregulation of *CASP9* via NF-κB-mediated FFL should be a molecular mechanism underlying the TNFα-promoted apoptosis of cancer cells induced by DOX. Clearly, *CASP9* is a new mediator of the NF-κB pro-apoptotic pathway in the examined cells. This result is similar to a recent finding that NF-κB plays a pro-apoptotic role by directly upregulating the pro-apoptotic target genes such as *CASP4* and *PUMA* [8,15,26]. Conversely, the cytoprotection by NF-κB is also due to transcriptional activations of a number of antiapoptotic proteins, such as c-FLIP, Bcl-2, Bcl-XL, cIAP2 and A1/Bfl-2 [26]. However, the mechanisms of NF-κB in apoptosis regulation remain controversial and poorly understood. The effect of NF-κB on apoptosis may depend on the balance of pro-apoptotic target genes and antiapoptotic target genes regulated by this TF.

Additionally, we also detected the expressions of the largely unknown miR1276 and found that it has an expression profile like that of its host gene *KLHL25* in various cell types, including HeLa, HepG2, ECa109, PC-9, A549, HL-7702, HCT116 and CaSKi cells (Figure S2). This finding is consistent with the other embedded miRNAs that are frequently transcribed in parallel with their host genes [33,34]. Like other miRNAs that post-transcriptionally repress the expression of their target genes, miRNA1276 was found to block the expressions of *CASP9* at both the mRNA level and protein level. Transcriptional and post-transcriptional regulations are two different methods for gene regulation. Thus, the regulation of *CASP9* by NF-κB-mediated FFL is performed through cross-layer coregulation. It has been reported that the expressions of many genes are controlled by the cross-layer coregulation of TFs and miRNAs [35,36,37]. Importantly, cross-layer coregulation has a higher specificity than intra-layer combinatorial regulation of TF–TF and miRNA–miRNA pairs [36]. We thus inferred that without the removal of the miR1276 inhibition of *CASP9*, NF-κB might be unable to increase *CASP9* expression. Therefore, our discovery of the cross-layer coregulation of *CASP9* by NF-κB-mediated FFL provides new insights into the molecular mechanism through which NF-κB realizes one of its important biological functions. 

In conclusion, we identified *CASP9* and miR1276 as direct target genes of NF-κB in TNFα-treated cells. *CASP9* was also identified as a target of miR1276. Thus, NF-κB cannot only directly regulate *CASP9* by itself but also indirectly regulates this gene through one of its target microRNAs, miR1276. Consequently, a coherent NF-κB-mediated FFL was established in this study by which a pro-apoptotic gene, *CASP9* was upregulated in TNFα-treated cells. Besides this, we found that TNFα promoted apoptosis induced by DOX. Upregulation of CASP9 protein was detected in this process. A specific CASP9 inhibitor repressed the TNFα promotion of apoptosis induced by DOX. Based on these investigations, the upregulation of *CASP9* by NF-κB-mediated FFL is proposed as a molecular mechanism underlying the TNFα promotion of apoptosis induced by DOX. This finding improves the understanding of a pro-apoptotic function of NF-κB under certain conditions.

## 4. Materials and Methods

### 4.1. ChIP-Seq Data Analysis

To characterize global BSs, we subjected TNFα-treated HeLa cells to chromatin immunoprecipitation followed by deep sequencing (ChIP-Seq) [16]. In this study, HeLa cells were treated with TNFα, and the NF-κB-bound chromatin was enriched with an anti-NF-κB RelA antibody (Ab). The enriched DNA was then sequenced with an Illumina Genome Analyzer II. The sequenced DNA reads were mapped to the human genome (hg19) using ELAND software with the default settings. The NF-κB-BSs (i.e., ChIP-Seq peaks) were found using the ChIP-Peak program with the following parameters: Window Width, 200; Vicinity Range, 200; Peak Threshold, 1; Count Cut-off, 10; and Repeat Masker, select. The NF-κB-BSs were assigned to genes according to their location in the genomic region from −100 kb upstream of the transcription start site (TSS) to +100 kb downstream of the transcription end site (TES) of the gene [38,39]. To identify the NF-κB DNA-binding sequences (i.e., κB) in the NF-κB-BSs, the DNA sequences of the NF-κB-BSs were uploaded to the TRANSFAC online server and searched with five high-quality NF-κB matrices (Accession No.: M00208, M00194, M00054, M00051, and M00052). The parameters were set as follows: the cut-off scores of the core match and matrix match were 0.75. A value of 0.75 was used as a threshold for identifying a sequence as NF-κB DNA-binding site [20].

### 4.2. Cell Culture and Treatment

HeLa cells and HepG2 cells were purchased from the China Center for Type Culture Collection, Chinese Academy of Sciences, Shanghai, China. The HeLa cells and HepG2 cells were cultured in Dulbecco’s modified Eagle’s medium (DMEM) supplemented with 10% fetal calf serum, 100 units/mL penicillin and 100 μg/mL streptomycin in 5% CO_2_ at 37 °C. To manipulate cellular NF-κB activity, the cells were treated with TNFα or siRNA. The cells subjected to the TNFα treatment were treated with 30 ng/mL TNFα (Sigma) for 1 h. The cells subjected to the siRNA treatment were first transfected with 30 nM NF-κB/RelA siRNA or Signal Silence Control siRNA (cell signaling technology) using Lipofectamine 2000 (Invitrogen) for 48 h and then were stimulated with 30 ng/mL TNFα for 1 h. To manipulate the cellular abundance of miR1276, the cells were transfected with a miR1276 mimic or a miR1276 expression plasmid (GenePharma) using Lipofectamine 2000. Negative control miRNA mimics and a GV268 vector without the miR1276 gene (GenePharma) were also transfected as controls. 

### 4.3. ChIP Expriment

A ChIP experiment was performed as in our previous ChIP-Seq studies [16]. The cells (5×10^7^) were treated with 30 ng/mL TNFα for 1 h, then crosslinked with 1% formaldehyde (Sigma) for 10 min at room temperature, and finally quenched with glycine (Sigma) at a final concentration of 125 mM. The cells were washed with ice-cold phosphate-buffered saline (PBS) and then incubated in ice-cold hypotonic lysis buffer for 10 min. The cells were then collected by centrifugation and resuspended in ice-cold nuclear lysis buffer. The cell nuclei were collected by centrifugation, and the chromatin was sheared into fragments between 200 to 500 bp by sonication. The sonicated products were then centrifuged at 13,000 rpm to collect supernatants (soluble chromatin). Fifty microliters of soluble chromatin (from 10^6^ cells) were diluted with 450 μL of chromatin dilution buffer. Then, 1.2 μg of Ab, such as anti-NF-κB RelA rabbit polyclonal Ab (ab7970; Abcam) or normal rabbit IgG (Sc-2027; Santa Cruz), was added to the diluted chromatin, and the mixture was incubated overnight at 4 °C. Additionally, 10% of the diluted chromatin was kept as an input. After the Ab reaction, the chromatin was incubated with Dynabeads Protein A (Invitrogen) at 4 °C for 2 h to capture the DNAs bound to NF-κB RelA. Subsequently, the beads were washed three times with low-salt washing buffer, high-salt washing buffer and Tris-EDTA (TE) buffer. The captured chromatin was eluted from the beads into 120 μL of elution buffer. RNase A (MBI Fermentas), Proteinase K (MBI Fermentas) and NaCl at final concentrations of 0.5 mg/mL, 0.2 mg/mL and 300 mM, respectively, were added to the eluted chromatin. The chromatin was incubated overnight at 65 °C for reverse crosslinking. Finally, DNA from the chromatin was extracted using a QIA-quick PCR purification kit (Qiagen). The constituents of all the solutions used in this and the other experiments performed this study are shown in the Supporting Information.

### 4.4. ChIP-qPCR

The ChIPed DNA was detected through quantitative polymerase chain reaction (qPCR). Ten percent of input DNA and 1.5 μL of anti-RelA Ab and normal rabbit IgG ChIPed DNAs were simultaneously detected by qPCR on an ABI StepOnePlus real-time PCR system (Applied Biosystems) using SYBR Green Real-time PCR Master Mix in a 20-μL reaction volume. The relative enrichment of anti-RelA ChIPed DNA to the normal IgG ChIPed DNA in the ChIP-qPCR detections was calculated as previously described [1]. In brief, the Ct of Ab-ChIPed DNA was normalized to that of the input DNA using the formula ΔCt_Ab_ = Ct_Ab_−(Ct_input_−3.322). The ChIP-qPCR signal of the Ab-ChIPed DNAs is represented as the percentage of input DNA. All of the primers used in the ChIP-qPCR detections are listed in Table S1. Student’s t-test was used to evaluate the statistical significance of the data obtained in this study.

### 4.5. Luciferase Reporter Assay

A minimal promoter (5′-TAGAGGGTATATAATGGAAGCTCGACTTCCAG-3′) was first inserted into the pGL4.10-basic vector (Promega) using the *Hind*III (5′) and *Nco*I (3′) sites (Takara). An NF-κB-BS in the *CASP9* promoter region from the genomic DNA of HeLa cells was amplified by PCR and inserted upstream of the minimal promoter using the *Kpn*I and *Hind*III sites. A construct with the mutated κB site was also constructed by fusion PCR. The PCR primers used for DNA cloning are shown in Table S1. All of the constructs were verified by DNA sequencing. The luciferase activity of the reporter constructs was quantified using a Dual-Luciferase Reporter Assay System (Promega) according to the manufacturer’s instructions. Briefly, the cells were cultured on a 24-well plate and co-transfected with 400 ng of firefly luciferase reporter construct and 40 ng of renilla luciferase control plasmid (pGL4.75). To manipulate the NF-κB activity in the transfected cells, the cells were stimulated with TNFα or co-transfected with RelA siRNA as described above. Twenty-four hours later, the cells were lysed, and the cell lysates were collected and subjected to a dual-luciferase reporter assay. The firefly luciferase signal was normalized to the renilla luciferase signal. 

The 3′ untranslated region (3′UTR) of *CASP9* containing the predicted miR-1276 binding site was amplified by PCR. The used primers were 5′-CCGCTCGAGCTGCCTTATCTTGCACCCCA-3′ and 5′-AAGGAAAAAAGCGGCCGCGGGACACAAGTCACTAGCCC-3′. The 279 bp products were inserted into the *Xho*I and *Not*I restriction site of the psiCHECK2 vector (Promega) and validated by sequencing. The mutant constructs were generated by mutation. Fragments were subcloned into the *Xho*I and *Not*I site in the psiCHECK2 vector. Herein, the psiCHECK2-*CASP9* 3′UTR or psiCHECK2-*CASP9* 3′UTR mutant reporter plasmids (300 ng) were co-transfected with miR1276 expressed plasmid or its negative control (Genechem, Shanghai) into HeLa cells using Lipofectamine 2000 (Invitrogen), in accordance with the manufacturer’s instructions. After 24 h, cells were lysed and reporter activity was assessed using the dual-luciferase reporter assay system (Promega, USA) in accordance with the manufacturer’s protocols. Renilla luciferase activity was normalized to firefly luciferase activity. 

### 4.6. Cell Viability and Apoptosis Detection

The cells were cultured for 24 h and then treated with 30 ng/mL TNFα, 5 µg/mL DOX (Hisun Pharmaceutical Co., Ltd.) or 30 ng/mL TNFα and 5 µg/mL DOX (hereafter denoted TNFα&DOX) for 12 h. Simultaneously, untreated cells were cultured and used as a control. The cell viability was then detected through cell counting kit-8 (CCK8) assay, the cell apoptosis was detected through DNA Laddering assay, microscopy and flow cytometry (FCM). For CCK8 assay, the HeLa cells cultured and treated in 96-well plates were detected by the enhanced cell counting kit-8 (Beyotime). A total of 10 μL CCK8 solution was separately added into each well and the cells were incubated at 37 °C under normal culture conditions for 4 h. The optical density (OD) at 450 nm wavelength was measured with a TriStar^2^ LB 942 multimode microplate Reader (Berthold). For microscopic observation, cells were first photographed with an inverted light microscope (IX51, Olympus). Cells were also fixed with 4% paraformaldehyde and stained with DAPI (Beyotime) for 30 min at room temperature. The stained cells were photographed with an inverted fluorescence microscope (IX51, Olympus). For FCM detection, all of the cells, including floating and adherent cells, were collected, fixed in ethanol and stained with propidium iodide (PI) solution as previously described [12,40]. The stained cells were detected and analyzed using an FCM system (NovoCyte). The percentage of cells with sub-G1 DNA content was considered a measure of the apoptotic rate of the cell population [12,40]. To assess the effect of caspase-9 activation on the apoptotic rate, HeLa cells were pretreated with or without a 50 µM aliquot of a cell permeable caspase-9 inhibitor (SCP0113; Sigma) for 30 min, then treated with TNFα, or DOX, or TNFα&DOX at the above-mentioned doses for 12 h, and finally detected through FCM. For DNA laddering assay, the total genomic DNA was extracted as reported previously [40]. Briefly, the collected HeLa cells were lysed in buffer containing 10 mM Tris-HCl (pH 8.0), 0.1 M EDTA and 0.5% SDS for 10 min. RNase A and proteinase K were supplemented subsequently and incubated overnight at 50˚C. The lysates were extracted with phenol and chloroform and centrifuged at 12,000 rpm for 5 min. The DNA precipitated with ethanol was retreated with RNase A. The extracted DNA was electrophoresed in 1.2% agarose gel and photographed.

### 4.7. Real-time Reverse Transcription-PCR

To detect the expression of protein-coding genes, the total RNA from the cells was extracted with Trizol (Invitrogen) and reverse transcribed into cDNA using a PrimeScript RT Master Mix (Takara) in a 20-μL system. The cDNA was used as the template for the detection of gene expression levels through real-time reverse transcription-PCR (qRT-PCR) with an ABI StepOnePlus real-time PCR system (Applied Biosystems) using a SYBR Green Real-time PCR Master Mix (Roche) in a 20-μL reaction volume. *GAPDH* was used as the endogenous control. To detect miR1276 expression, the total RNA from the cells was isolated with Trizol (Invitrogen) and reverse transcribed using a BU-Script RT Kit (Biouniquer, Nanjing) in a 20-μL system. One microliter of reverse-transcribed product was detected by qRT-PCR with an ABI StepOnePlus real-time PCR system (Applied Biosystems) using a SYBR Green Real-time PCR Master Mix (Roche) in a 20-μL reaction volume. The level of snRNA *U6* expression was used for the normalization of miR1276 expression. All qRT-PCRs were performed for at least three independent experiments, and each independent experiment included three replicates. The specificity of the PCR amplifications was determined through melting curve analysis. The expression level of the detected genes is presented as the relative quantification (RQ) and calculated using the comparative Ct method. All primers used for the qRT-PCR detections are listed in Table S1.

### 4.8. Western Blotassay

The total protein from the cells was isolated with cell lysis buffer. The nuclear extract from the cells was isolated using a Nuclear Extract Kit (ActiveMotif) according to the manufacturer’s instructions. The protein extract was separated by sodium dodecyl sulfate-polyacrylamide gel electrophoresis (SDS-PAGE) and transferred to a polyvinylidene difluoride (PVDF) membrane (Millipore). The membrane was first blocked with blocking buffer containing 5% (w/v) skim milk and then incubated with antibody dilution buffer containing various primary antibodies, such as rabbit anti-RelA/RelA Ab (ab7970; Abcam), rabbit anti-CASP9 Ab (Boster), mouse anti-TATA-binding protein (TBP) Ab (Abcam) and mouse anti-actin Ab (Beyotime). Subsequently, the membrane was washed three times with washing buffer and incubated with antibody dilution buffer containing the different secondary antibodies, such as IRDye 800CW goat anti-mouse IgG and IRDye680CW goat anti-rabbit IgG (Li-Cor). The membrane was washed three times with washing buffer and imaged with an Infrared Imaging System (Li-Cor). The signal intensity of the protein bands on the WB assay images was quantified with ImageJ software. The target protein signal was normalized to that of the loading control protein (actin or TBP).

### 4.9. miRNA Target Prediction

Three online programs, namely DIANA-microT-CDS [41], miRWalk [42] and TargetScan [43], were used for the prediction of miR1276 target genes. When the DIANA-microT-CDS program was used to predict the binding of miR1276 to *CASP9* mRNA, the miTG Score Threshold was set to 0.75. The parameters used with miRWalk software were as follows: transcript, the longest transcript of genes; minimum seed length, 7 nt; and cut-off of *p* value, 0.05. When TargetScan was used to predict the binding of miR1276 to *CASP9* mRNA, the *CASP9* mRNA sequence was directly submitted to the server for the prediction of the potential miRNA binding sites on its 3′UTR using the default parameters.

## Figures and Tables

**Table 1:** Nuclear factor-κB binding sites (NF-κB-BSs) assigned to *CASP9* and their κB contents.

BSs	FE	κB Number	Location
BS1	20.579	44	3′ region
BS2	21.720	23	3′ region
BS3	21.236	55	3′ region
BS4	34.436	14	3′ region
BS5	30.491	8	3′ region
BS6	21.982	8	3′ region
BS7	22.187	21	3′ region
BS8	21.805	3	3′ region
BS9	20.638	31	3′ region
BS10	27.507	17	5′ region
BS11	21.918	19	5′ region
Average	24.045	22.091	

**Table 2:** NF-κB-BSs assigned to *miR1276/KLHL25* and their κB contents.

BSs	FE	κB Number	Location
BS1	22.87	3	3′ region
BS2	21.869	4	3′ region
BS3	20.815	10	3′ region
BS4	32.358	28	3′ region
BS5	20.386	68	3′ region
BS6	21.175	77	Internal
BS7	25.373	55	Internal
BS8	23.653	30	Internal
BS9	28.99	35	5′ region
BS10	25.738	4	5′ region
BS11	29.767	2	5′ region
Average	24.818	28.727	

**Table 3:** Prediction of *CASP9* as a target of miR1276 using three online programs.

	miRWalk	TargetScan	DIANA-microT-CDS
Binding type	7 mer	7 mer-1A/7 mer-m8	7 mer/6 mer/6 mer/7 mer/6 mer/6 mer/7 mer(CDS)
Potential binding site	1	1	7 (6 in 3′ UTR +1 in CDS)
Region	3′ UTR	3′ UTR	3′ UTR and CDS
Confidence	*P* value = 0.041	Context + +score = −0.11	miTG core = 0.766

## Supplementary Materials

Supplementary Materials can be found at https://www.mdpi.com/1422-0067/21/7/2290/s1.

## Abbreviations

BSs	NF-κB-binding sites
CASP	Caspase
cCASP9	cleaved CASP9
DOX	doxorubicin
fCASP9	full-length CASP9 protein
TF	transcription factor
FFL	feed-forward loop
RQ	relative quantification
tCASP9	total CASP9
ChIP-Seq	Chromatin Immunoprecipitation-Sequencing
