# Supplementary material for: Upregulation of CASP9 through NF-κB and Its Target MiR-1276 Contributed to TNFα-Promoted Apoptosis of Cancer Cells Induced by Doxorubicin
Source: Int J Mol Sci. 2020 Mar 26;21(7):2290. doi: 10.3390/ijms21072290 (PMC7177739; doi:10.3390/ijms21072290)
Supplement: Supplementary file 1 [file ijms-21-02290-s001.pdf]

# Supplementary materials

## Cell culture

HeLa, HepG2, ECa109, PC-9, A549, HL-7702, HCT116 and CaSKi cells were purchased from the China Center for Type Culture Collection, Chinese Academy of Sciences, Shanghai, China. All these cells were cultured in Dulbecco's modified Eagle's medium (DMEM) supplemented with 10% fetal calf serum, 100 units/mL penicillin and 100 μg/mL streptomycin in 5% $CO_2$ at 37 °C.

## Chemicals

The Dulbecco's modified Eagle's medium (DMEM), fetal calf serum, penicillin, streptomycin, Lipofectamine 2000, Trizol reagent, and Dynabeads Protein A were purchased from Invitrogen. DOX was purchased from HiSun Pharmaceutical. TNF$\alpha$, cell permeable caspase-9 inhibitor (SCP0113), paraformaldehyde, sodium dodecyl sulfate (SDS), phenylmethanesulfonyl fluoride (PMSF), RNase A, Proteinase K. TritonX-100, and Tween 20 were purchased form Sigma. The pGL4.10-basic vector and Dual-Luciferase Reporter Assay System were purchased from Promega. The NF-κB/RelA siRNA and the Signal Silence Control siRNA were purchased from Cell signaling Technology. The miR1276 mimic, a negative control miRNA mimic, the miR1276 expression plasmid (GV268 vector with mir1276 gene), and a GV268 vector without mir1276 gene were purchased from Genepharma (Shanghai). The anti-NF-κB RelA rabbit polyclonal antibody (ab7970), and the mouse anti-TATA binding protein (TBP) antibody were purchased from Abcam. The normal rabbit IgG (Sc-2027) was purchased from Santa Cruz. The rabbit anti-CASP9 antibody was purchased from Boster. The mouse anti-actin antibody, DAPI, and acrylamide solution were purchased from Beyotime (Nanjing, China). The IRDye 800CW Goat anti-mouse IgG and the IRDye680CW Goat anti-rabbit IgG were purchased from Li-Cor. The QIA-quick PCR purification kit was purchased from QIAGEN. The SYBR Green Real-time PCR Master Mix and protease inhibitor cocktail (PIC) was purchased from Roche. The enzymes including Hind III, NcoI, and KpnI and the PrimeScript RT Master Mix were purchased from Takara. The Nuclear Extract Kit was purchased from Active motif. The BU-Script RT Kit was purchased from Biouniquer (Nanjing, China). The PVDF membrane was purchased from Millipore. The PCR primers (Table S1) were manufactured by the GeneScript (Nanjing, China). The skim milk was purchase form supermarket.

## Solutions and constituents

All other solutions were prepared by ourselves, including the phosphate buffered saline (PBS), hypotonic lysis buffer, Nuclei lysis buffer, chromatin dilution buffer, low-salt washing buffer, high-salt washing buffer, Tris-EDTA (TE) buffer, elution buffer, Propidium Iodide (PI) solution, and cell lysis buffer. The constituents of these solutions are as follows.

**FCM assay**

[1]. Propidium Iodide (PI) solution: 0.1% TritonX-100, 100 μg/ml RNase, 50 μg/ml PI.

**DNA laddering assay**

Cell Lysis solution: 10 mM Tris-HCl (pH 8.0), 0.1 M EDTA, and 0.5% SDS.

**ChIP assay**

[1]. Hypotonic lysis buffer: 0.2% NP-40, 10 mM NaCl, 10 mM Tris-HCl, pH8.0; Add 10 μL PIC and 10 μL 0.1 M PMSF per ml just before use.

[2]. Nuclei lysis buffer: 1% SDS, 10 mM EDTA, pH8.0, 50 mM Tris-HCl, pH8.0; Add 10 μL PIC and 10 μl 0.1M PMSF per mL just before use.

[3]. Chromatin dilution buffer: 1.1% Triton X-100, 0.01% SDS, 1.2 mM pH8.0 EDTA, 167 mM NaCl, 16.7 mM Tris-HCl, pH8.0; Add 10μL PIC and 10 μL 0.1M PMSF per mL just before use.

[4]. Low salt washing buffer: 0.1% SDS, 1% Triton X-100, 2 mM pH8.0 EDTA, 150 mM NaCl, 20 mM Tris-HCl, pH8.0; Add 10 μL PIC and 10 μL 0.1 M PMSF per mL just before use.

[5]. High-salt washing buffer: 0.1% SDS, 1% Triton X-100, 2 mM pH8.0 EDTA, 500 mM NaCl, 20 mM Tris-HCl, pH8.0; Add 10 μL PIC and 10 μL 0.1 M PMSF per mL just before use.

[6]. Elution buffer for ChIP: 1% SDS, 50 mM Tris-HCl, 10 mM EDTA

[7]. TE buffer: 1 mM EDTA pH 8.0, 10 mM Tris-HCl, pH 8.0

**Western blot assay**

[1]. Cell lysis buffer: 50 mM Tris-HCl, pH8.0, 150 mM NaCl, 1% TritonX-100, and 100 μg/ml PMSF. This buffer was used to extract total protein from cells.

[2]. 0.01 M PBS solution: 137 mM NaCl, 2.7 mM KCl, 4.3 mM $Na_2HPO_4.7H_2O$, 1.4 mM $KH_2PO_4$

[3]. Washing buffer: 0.01 M PBS, 0.05% (v/v) Tween 20

[4]. Blocking buffer: 0.01 M PBS, 5% (w/v) skim milk, 0.05% (v/v) Tween 20

[5]. Antibody dilution buffer: 0.01 M PBS, 5% (w/v) BSA, 0.05% (v/v) Tween 20

**Table S1.** Primers used in this study

| Usage | Gene | Primers (5' to 3') | |
|---|---|---|---|
| RT | snRNAU6 | AACGCTTCACGAATTTGCGT | |
| | miR1276 | TCGTATCCAGTGCAGGGTCCGAGGTATTCGCACTGGATACGACTGTCTCC | |
| cDNA | STAT5A | GGTGAGATCCTGAACAACTGC | TGAACTTCTCCTCTGTCACGG |
| | NF-κB2 | CGTGAAAGACCCTCCTGTTC | AGAGCGAGATCCGGAGTTG |
| | RELB | CCGCCAGATTGCCATTGTGTTC | TCTTCCGCCGTTTGCTCTCG |
| | IL7R | GCCAATGACTTTGTGGTGAC | CACATGCGTCCATTTGTTTT |
| | IL6 | TGGGCACAGAACTTATGTTG | TTGAGGTAAGCCTACACTTTCC |
| | CASP9 | CGAACTAACAGGCAAGCAGCAAAG | AGAGCACCGACATCACCAAATCC |
| | KLHL25 | TTAGAAAACTGATAAAAGCAACACA | TGGGGTTGCTTCATTGAGAT |
| | GAPDH | ATTTGGTCGTATTGGGCG | CTCGCTCCTGGAAGATGG |
| | miR1276 | TAAAGAGCCCTGTGGAG | GTGCAGGGTCCGAGGT |
| | snRNAU6 | CTCGCTTCGGCAGC ACA | AACGCTTCACGAATTTGCGT |
| gDNA | STAT5A | GTCCTGGTGTGAGAAGTTGGC | GGCTGAGATAATGTCCGTGATGG |
| | IL7R | TTCCAAGTTCAAGCTCCACA | TTCCCTCAGTTTAGCAGAAGATA |
| | RELB | GATAGAGCCCACTGTATCTG | GCCACCATCAACCCAATA |
| | CASP9 | CGGCTGTCTGTGTGTGGCTTTAGAC | TCCCGGTTGCTGTAGTACCATCCCT |
| | CASP9 | GCTTGTCCCCTCCTCTTTCCCCTCC | CAGCTCCGTGCTCCAGTTAGTTCGC |
| | CASP9 | ATATCTCTGCTTATGTTCCCAG | CAAACATAGTTACCTTGGTAGTAT |
| | KLHL25 | AATACGAGGTTGGTGCTGATGGG | ATGAAGTTGGGTGGTTTGAGGGA |
| | KLHL25 | AAACTGACGAGATTAGAGCTAGCAA | GAGTAGGATGGGGATCTCACTTTAC |
| | KLHL25 | TATCTAAAGAAGCTGCAGCCACTCATC | ATTGCCAGAGGTGTGGGAGG |
| pCON | CASP9 | CGGGGTACC CGGTAGGAGCGGAAACAC | CCCAAGCTTCGTCTACTGGGCGAATCTA |
| | CASP9-κBM | CGGGGTACCCGGTAGGAGCTCTAACCTCTTCC TGGATCGAGGAGGTCC | CCCAAGCTTCGTCTACTGGGCGAATCTA |

Note: RT, primers used to the reverse transcription of microRNA; cDNA, primers used to qRT-PCR detections of complementary DNA (cDNA); gDNA, primers used to qPCR detections of the ChIPed genomic DNA (gDNA); pCON, primers used to the plasmid construction (pCON).

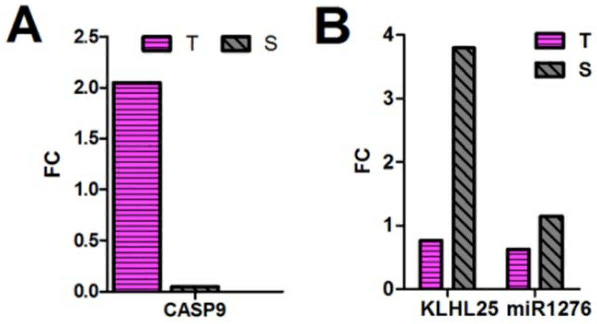


**Fig.S1. NF-κB regulation of *CASP9*, *KLHL25*, and *miR1276*.** TNF$\alpha$ was used to induce NF-κB activity and RelA siRNA was used to block NF-κB activity in the HeLa cells. The HeLa cells treated by the Signal Silence Control siRNA were used as negative controls. GeneChip and miR-Seq were performed with the control siRNA-treated HeLa cells and NF-κB activity-operated HeLa cells for detecting mRNA and miRNA expression profile, respectively. The transcripts of *CASP9* was increased by the TNF$\alpha$ treatment but decreased by the RelA siRNA treatment. However, the transcripts of *KLHL25* and *miR1276* were decreased by the TNF$\alpha$ treatment but increased by the RelA siRNA treatment. The fold change (FC) of *CASP9* and *KLHL25* transcripts was detected by GeneChip (A) and FC of *miR1276* transcripts was detected by miR-Seq (B). T, TNF$\alpha$; S, siRNA.

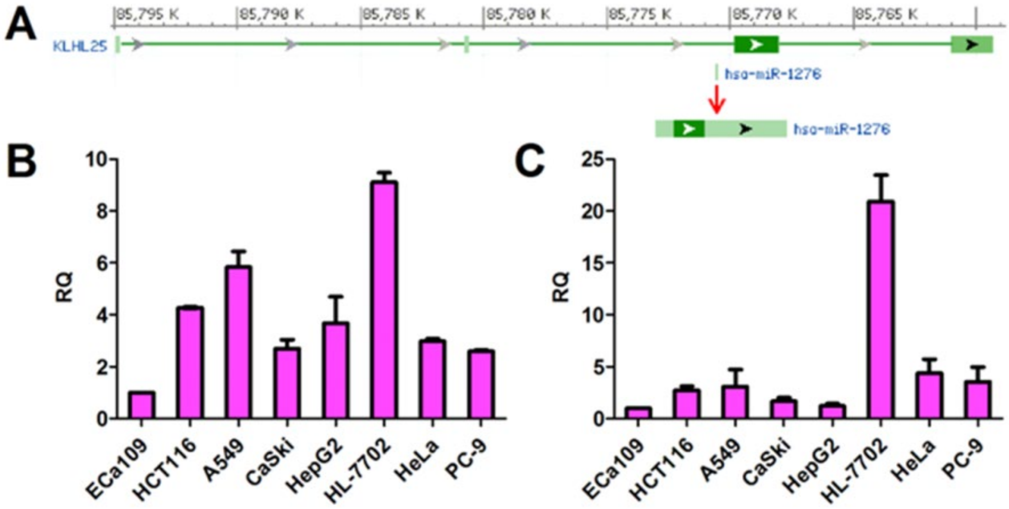


**Fig.S2. Genomic location of *KLHL25* and *miR1276* genes and their expression in multiple cells**. A. Genomic location of *KLHL25*/*miR1276* genes on Chromosome 15 in the view of human genome assembly (GRCh38). The red arrow points to the enlarged *miR1276* gene. B. Expression of *KLHL25* in eight cell lines detected by qRT-PCR. C. Expression of *miR1276* in eight cell lines detected by qRT-PCR. RQ, relative quantification.

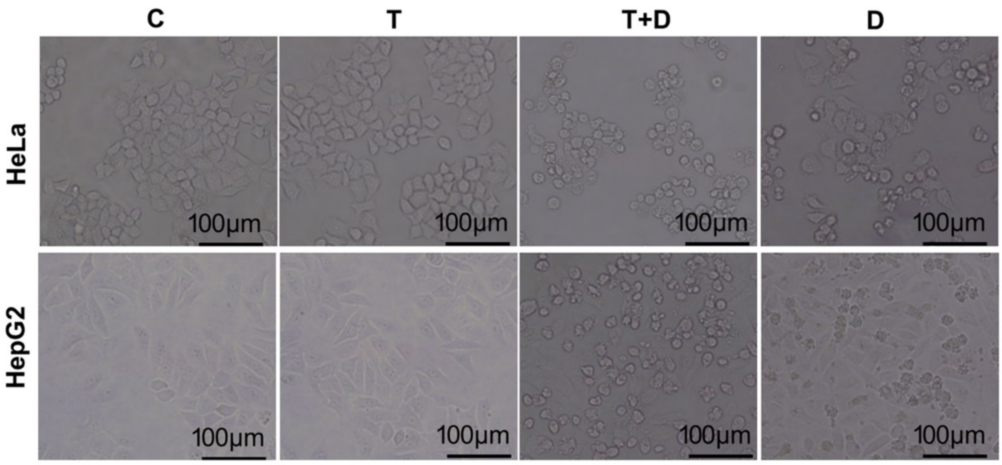


**Fig.S3. DOX induced apoptosis of HeLa and HepG2 cells with or without TNF$\alpha$-cotreatment**.
The morphological characteristics of apoptotic cells were observed by light microscopes.
